# A triterpenoid from wild bitter gourd inhibits breast cancer cells

**DOI:** 10.1038/srep22419

**Published:** 2016-03-01

**Authors:** Li-Yuan Bai, Chang-Fang Chiu, Po-Chen Chu, Wei-Yu Lin, Shih-Jiuan Chiu, Jing-Ru Weng

**Affiliations:** 1 Division of Hematology and Oncology, Department of Internal Medicine , China Medical University Hospital, Taichung 404, Taiwan; 2 Cancer Center, China Medical University Hospital, Taichung 404, Taiwan; 3 College of Medicine, China Medical University, Taichung 404, Taiwan; 4 Institute of Biological Chemistry, Academia Sinica, Taipei 115, Taiwan; 5 Institute of Basic Medical Sciences, College of Medicine, National Cheng Kung University, Tainan 701, Taiwan; 6 Department of Pharmacy, Kinmen Hospital, Kinmen 891, Taiwan; 7 School of Pharmacy, Taipei Medical University, Taipei 110, Taiwan; 8 Department of Biological Science and Technology, China Medical University, Taichung 404, Taiwan

## Abstract

The antitumor activity of 3β,7β,25-trihydroxycucurbita-5,23(*E*)-dien-19-al (TCD), a triterpenoid isolated from wild bitter gourd, in breast cancer cells was investigated. TCD suppressed the proliferation of MCF-7 and MDA-MB-231 breast cancer cells with IC_50_ values at 72 h of 19 and 23 μM, respectively, via a PPARγ−independent manner. TCD induced cell apoptosis accompanied with pleiotrophic biological modulations including down-regulation of Akt-NF-κB signaling, up-regulation of p38 mitogen-activated protein kinase and p53, increased reactive oxygen species generation, inhibition of histone deacetylases protein expression, and cytoprotective autophagy. Together, these findings provided the translational value of TCD and wild bitter gourd as an antitumor agent for patients with breast cancer.

Being the most common cancer for female, breast cancer affects over one million women per year worldwide^1^. The American Cancer Society estimates more than 235,000 invasive breast cancer patients with 40,430 death in the United States in 2014^2^. In Taiwan, invasive breast cancer is also the most common cancer for female, with an incidence of 904 per million women and a mortality rate of 254 per million women in 2012. Although the much progress beyond the chemotherapy and hormonal therapy, including strategies against human epidermal growth factor receptor and against angiogenesis, the 5-year overall survival rate of patients with metastatic breast cancer is around 22%. This unsatisfied feature highlights the necessity of new therapeutic strategies.

Phytochemicals have provided diverse pharmacological effects including anti-tumor, anti-inflammation, anti-oxidant and anti-bacteria^3^. Some of the phytochemicals are widely evaluated their applications in clinical use^4^. For example, taxol analogues and, vinca alkaloids have been used for chemotherapy for decades^4^,^5^. In addition to medicinal plants, vegetables and foods are part of the sources of valuable phytochemicals. Wild bitter gourd, a vegetable, is also used as folk medicine because of its anti-diabetic, anti-inflammatory, and chemopreventive effects. Triterpenoids, fatty acids, proteins, and carotenoids are rich in wild bitter gourd which is responsible for the activity^6^,^7^,^8^,^9^. Interestingly, some triterpenoids showed anti-tumor activity in various cancer cells including breast cancer^8^,^10^.

There are several triterpenoids isolated from the crude extract of wild bitter gourd^11^,^12^. We previously isolated and purified a triterpenoid, 3β,7β-dihydroxy-25-methoxycucurbita-5,23-dien-19-al, which induced breast cancer cells apoptosis, in part, through modulating peroxisome proliferator-activated receptor (PPAR)γ−targeted gene products^12^. To further explore and validate the anti-tumor effect of wild bitter gourd, in the present study, we investigated the efficacy and underlying mechanisms of another triterpenoid, 3β,7β,25-trihydroxycucurbita-5,23(*E*)-dien-19-al (TCD; structure shown in Fig. 1A) against breast cancer cells. To the best of our knowledge, we demonstrated for the first time that TCD induced apoptotic death in breast cancer cells through Akt-NF-κB signaling, reactive oxygen species (ROS) production, and histone deacetylase inhibition.

## Results

### TCD inhibits the proliferation of breast cancer cells

**Figure 1:**
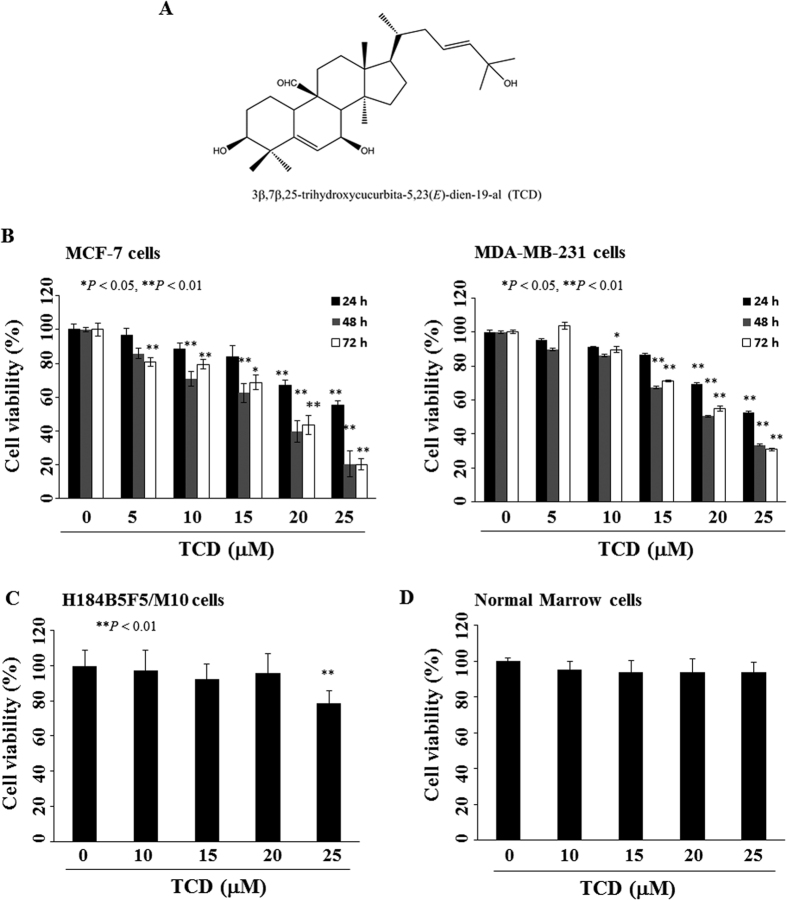
Antiproliferative effects of 3β,7β,25-trihydroxycucurbita-5,23(*E*)-dien-19-al (TCD) in breast cancer cells, normal human breast epithelial cells, and primary bone marrow cells. (**A**) The chemical structure of TCD. (**B**) Dose-dependent suppressive effects of TCD on the viability of MCF-7 and MDA-MB-231 breast cancer cells. Cells were treated with TCD at indicated concentrations in 5% fetal bovine serum (FBS)-supplemented DMEM/F12 medium for 24, 48, and 72 h, and cell viability was determined by MTT assays. *Points*, mean; *bars*, S.D. (n = 6). *P < 0.05; **P < 0.01. (**C**) Non-tumorgenic human breast epithelial cells (H184B5F5/M10) were treated with TCD for 24 h, and cell viability was determined by MTT assays. *Points*, mean; *bars*, S.D. (n = 6). (**D**) Normal bone marrow nuclear cells were incubated with TCD for 24 h. The cell viability was assessed using flow cytometry with annexin V-FITC and PI staining (n = 3).

We first examined the antiproliferative effect of TCD in two breast cancer cell lines, MCF-7 and MDA-MB-231, using 3-(4,5-dimethylthiazol-2-yl)-2,5-diphenyltetrazolium bromide (MTT) assay (Fig. 1B). TCD induced a concentration- and time-dependent suppressive effect on cell proliferation with an IC_50_ value of 19 and 23 μM at 72 h for MCF-7 and MDA-MB-231 cells, respectively. Additionally, both the non-tumorgenic human breast epithelial cell line (H184B5F5/M10) and the normal bone marrow nucleated cells were less sensitive to TCD with IC_50_ values > 25 μM (Fig. 1C,D).

### TCD induces breast cancer cells a caspase-dependent apoptosis without PPARγ activation

**Figure 2:**
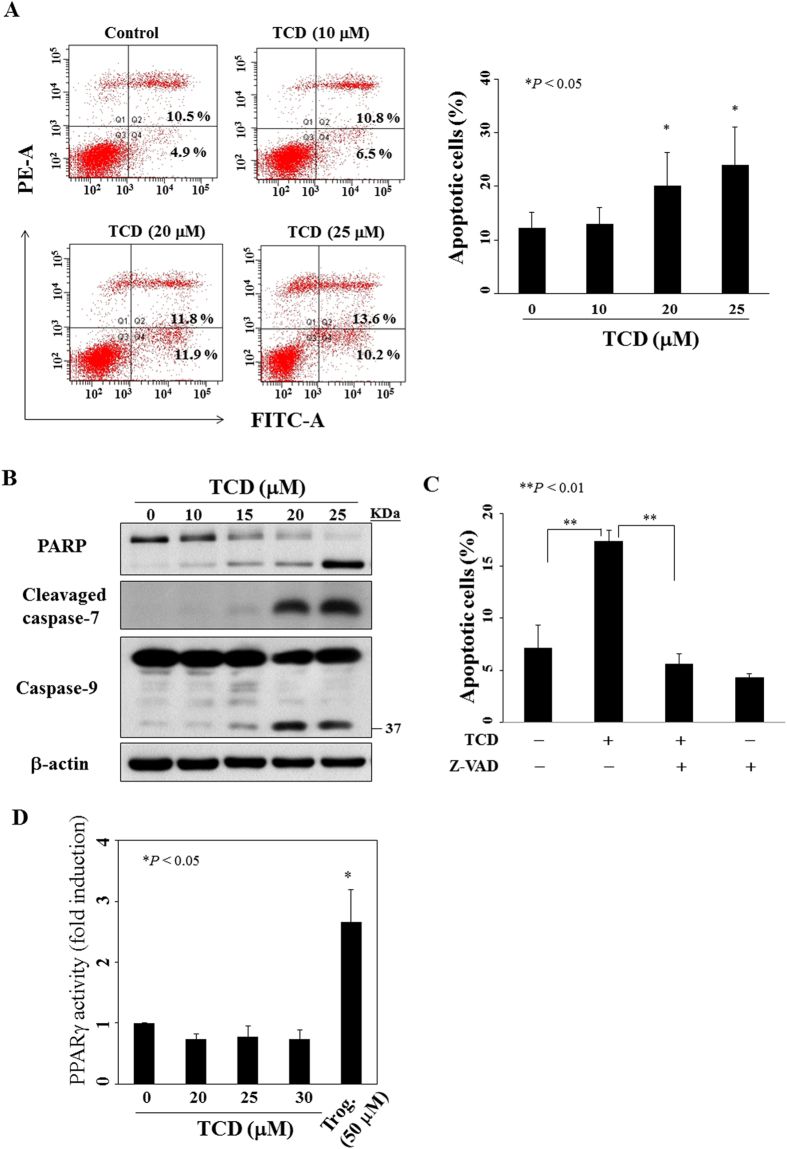
TCD induces apoptosis without increment of PPARγ activity in MCF-7 cells. (**A**) Left panel shows the dose-dependent effect of TCD on annexin V/PI staining of MCF-7 cells at 72 h. Right panel is the histogram which is representative of three independent experiments. Points, mean; bars, S.D. (n = 3). *P < 0.05 compared to the control group. (**B**) The dose-dependent effect of TCD on PARP cleavage, caspase-7 activation, and caspase-9 activation in MCF-7 cells after 72 h exposure. (**C**) The percentage of apoptotic cells of MCF-7 which was treated with DMSO, or TCD (20 μM) for 72 h with or without pre-treatment of 20 μM Z-VAD(OMe)-FMK (Z-VAD) (n = 3). The apoptotic cells were cells with annexin V positive cells in flow cytometric analysis. **P < 0.01. (**D**) Effect of TCD on PPARγ activation in MCF-7 cells. Cells were transfected with PPRE x3-TK-Luc and Renilla plasmids for 24 h before treatment with TCD for 24 h. Data are expressed as the fold change of PPARγ activity in each situation compared to the control. Troglitazone (Trog.) was used as positive control. Values are means ± S.E.M. of three independent experiments. *P < 0.05 compared to the control group.

To determine whether the reduced cell viability was attributed to apoptosis, we performed annexin V/propidium iodide (PI) staining in cells treated with TCD for 72 h. Flow cytometric analysis demonstrated that TCD increased the percentage of annexin V positive cells in a dose-dependent manner (Fig. 2A). The presence of apoptosis was further supported by PARP cleavage, increased cleavage of caspase-7 and caspase-9 in MCF-7 cells treated with TCD for 72 h (Fig. 2B).

Next, we investigated the role of caspase in TCD-induced apoptosis. Pre-treatment with Z-VAD(OMe)-FMK, a pan-caspase inhibitor, rescued TCD-induced apoptosis in MCF-7 cells (Fig. 2C). Similarly, TCD also induced a caspase-dependent apoptosis in MDA-MB-231 cells (Fig. S1A–D).

Some investigations and our previous study indicated that some triterpenoids from wild bitter gourd activated PPARγ which contributed to part of the anti-tumor activity of wild bitter gourd^10^,^12^. To clarify the role of PPARγ activation in TCD-induced apoptosis of breast cancer cells, accordingly, MCF-7 cells were transfected with pPPRE-TK-Luc plasmid which luciferase activity increased with increased PPARγ activity. Cells were then exposed to TCD at different concentrations or the positive control troglitazone (50 μM) for 24 h to assess the ability of TCD to transactivate PPARγ (Fig. 2D). Although the PPARγ agonist troglitazone induced activation of the reporter gene, TCD lacked the appreciable activity in PPARγ transactivation. A similar phenomenon was observed in TCD-treated MDA-MB-231 cells (Fig. S1E). These data suggested that PPARγ activation was not involved in TCD-induced cell apoptosis of MCF-7 and MDA-MB-231.

### TCD modulates Akt-NF-κB signaling and inhibits histone deacetylases (HDACs) activities

**Figure 3:**
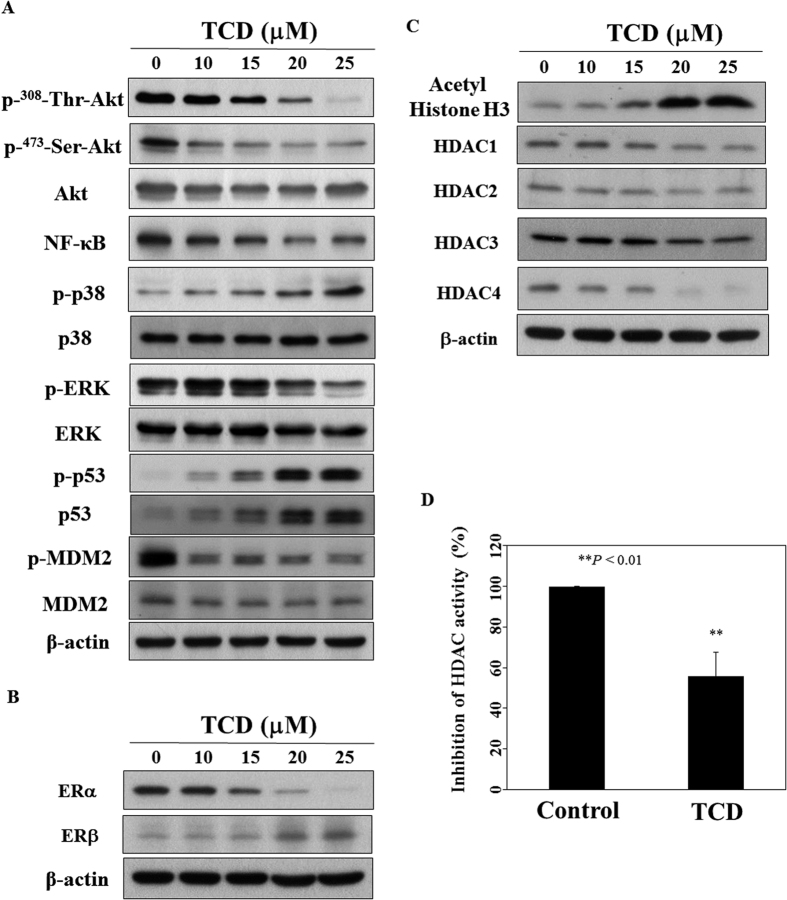
Western blotting analysis of TCD on apoptosis-related biomarkers and HDAC activity in MCF-7 cells. (**A**) Dose-dependent of TCD on the phosphorylation and protein expression of Akt, NF-κB, p38 MAPK, ERK, p53 and MDM2. Cells were treated with TCD in 5% FBS-supplemented DMEM/F12 medium for 72 h. (**B**) Effects of TCD on the expression of ERα and ERβ. (**C**) Effects of TCD on acetyl Histione H3 and HDACs expression. (**D**) TCD (20 μM) inhibited HDAC activity in MCF-7 cells. After treated with DMSO or TCD for 72 h, nuclear extract was used to assess HDAC activity as described in Materials and Methods. (n = 3). **P < 0.01.

It has been reported that triterpenoids isolated from wild bitter gourd and bitter gourd crude extracts down-regulate Akt and NF-κB, and inhibit HDAC activity^13^,^14^,^15^. Our data suggested that TCD down-regulated phosphorylated-Akt and its downstream target NF-κB as well in MCF-7 (Fig. 3A) and MDA-MB-231 cells (Fig. S2A). Moreover, the dephosphorylation of Akt was accompanied by parallel decrease in phosphorylated ERK and increase in the phosphorylation of p38 mitogen-activated protein kinase (MAPK). Because p38 MAPK signaling activated p53 in breast cancer cells^16^, we next examined the expression of p53. Western blotting demonstrated that TCD up-regulated p53 phosphorylation and protein expression (Fig. 3A, Fig. S2A). Compatible with the up-regulation of p53 protein expression was the parallel down-regulation of the phosphorylated MDM2, which is an E3 ubiquitin ligase targeting p53 for proteasomal degradation^17^.

It has been reported that ERβ antagonized ERα-induced cell proliferation^18^. Additionally, HDACs participate in gene regulation mediated by nuclear receptors including ER^19^. For example, HDAC1 interacts with ERα and suppresses its transcriptional activity *in vitro* and *in vivo*, and HDAC inhibitors can reverse the resistance of antiestrogen therapies in breast cancer^20^,^21^. Therefore, we evaluated the influence of TCD on the expression of estrogen receptor and HDACs in breast cancer cells. The result showed that TCD increased the ratio of ERβ to ERα expression in MCF-7 cells (Fig. 3B). In ERα-null MDA-MB-231 cells, TCD increased the expression of ERβ (Fig. S2A). Interestingly, we found that TCD induced HDAC inhibition as evidenced by increased acetyl histone H3 expression in a concentration-dependent manner as well as the HDAC activity in MCF-7 cells (Fig. 3C,D). Compatible with the finding was the down-regulation of HDAC1, HDAC2, HDAC3 and HDAC4 protein expression (Fig. 3C). In MDA-MB-231 cells, TCD did not inhibit HDAC activity significantly which reflected the conservation of HDAC protein expression after TCD treatment (Fig. S2C). The difference of HDAC activity inhibition by TCD between MCF-7 and MDA-MB-231 suggested a cell type-specific action.

### TCD induces apoptosis through ROS generation

**Figure 4:**
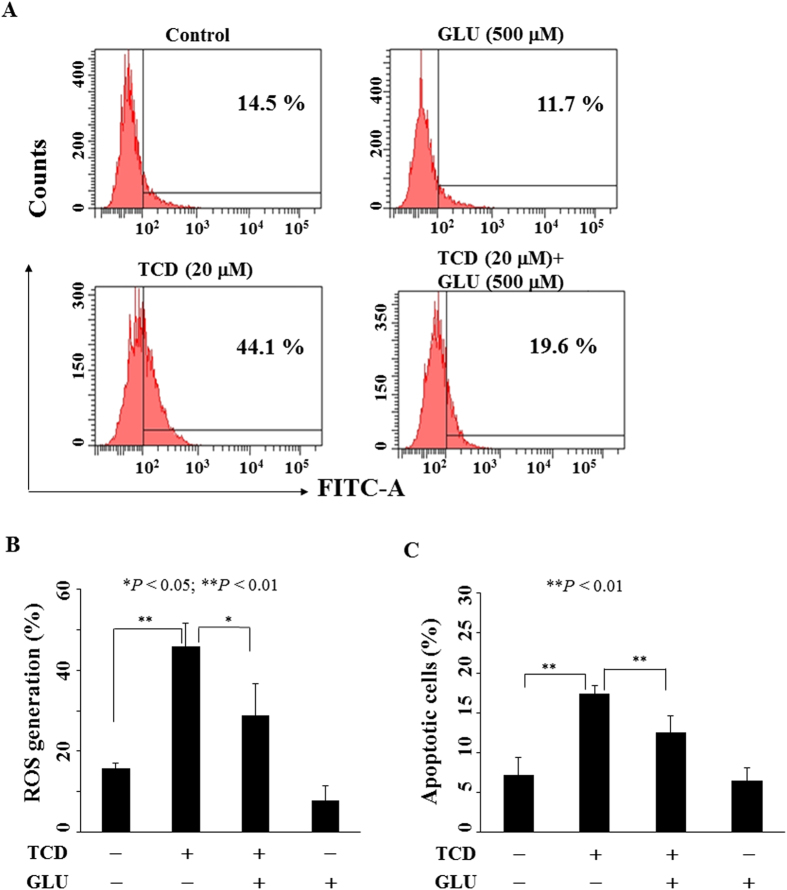
TCD induced reactive oxygen species (ROS) generation in MCF-7 cells. (**A**) Cells were treated with DMSO or 20 μM TCD with or without glutathione (GLU) for 72 h. (**B**) Histogram of ROS production in MCF-7 cells treated with DMSO or TCD with or without GLU for 72 h. (n = 3). *P < 0.05; **P < 0.01. (**C**) Pre-treatment with 500 μM glutathione (GLU) rescued partially the apoptosis induced by TCD (20 μM) for 72 h (n = 3).

Increased ROS has been reported to be responsible for the anti-tumor activity of some phytochemicals including curcumin, resveratrol, and triterpenoids^22^,^23^,^24^, thus, we examined the ROS generation in MCF-7 cells treated with TCD (Fig. 4A). TCD increased ROS generation by 29% compared to control group (44% vs. 15%) in MCF-7 cells. Pre-treatment with an antioxidant glutathione could significantly reverse TCD-induced ROS generation (Fig. 4B) and apoptosis (Fig. 4C). Similarly, TCD induced ROS generation which was rescued by glutathione pre-treatment in MDA-MB-231 cells (Fig. S3).

### TCD induces protective autophagy but not autophagic cell death in MCF-7

**Figure 5:**
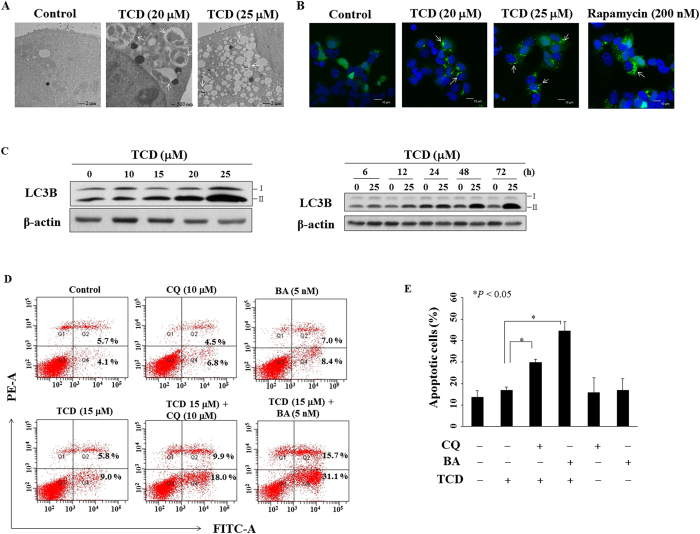
TCD induced autophagy in MCF-7 cells. (**A**) Electron microscopic analysis of autophagosome formation (arrow) in vehicle- or TCD-treated MCF-7 cells. Magnification, 12,000× (**B**) Fluorescent confocal microscopic analysis of TCD-induced autophagosome formation in MCF-7 cells ectopically expressing GFP-LC3. MCF-7 cells transfected with GFP-LC3 plasmids were treated with DMSO, 20 or 25 μM TCD, or 200 nM rapamycin for 30 min and then fixed by 3.7% paraldehyde and examined by confocal microscopy. The arrow indicates LC3-positive puncta in cells. (**C**) Dose- and time dependent effects of TCD on the expression of LC3B-II. (**D**) The apoptosis of MCF-7 cells induced by TCD could not be rescued by two autophagy inhibitors, chloroquine (CQ) and bafilomycin A1 (BA). MCF-7 cells were treated with DMSO, or TCD (15 μM) with or without 10 μM CQ or 5 nM BA for 72 h. (**E**) Histogram of autophagy inhibitors to increase the apoptotic cell percentage of MCF-7 induced by TCD. MCF-7 cells were treated with DMSO, or TCD (15 μM) with or without treatment of 10 μM CQ or 5 nM BA for 72 h (n = 3). *P < 0.05.

In light of recent reports indicating that triterpenoids induced autophagy in breast cancer cells^12^,^24^, we examined the effect of TCD on autophagy induction in MCF-7 cells. Using transmission electron microscopy, we found that exposure of MCF-7 cells to TCD at 20 or 25 μM for 24 h led to autophagosomes formation (arrow) in the cytoplasm (Fig. 5A). To validate this finding, we transiently transfected with GFP-tagged LC3 (GFP-LC3) in MCF-7 cells which were then exposed to TCD or control group for 24 h (Fig. 5B). Compared with DMSO-treated cells, TCD-treated cells exhibited more accumulation of LC3-positive puncta (arrow) in the cytoplasm under confocal fluorescence imaging similar to rapamycin-treated cells (positive control). The conversion of lipidated form of LC3B-I to LC3B-II is granted as an autophagosomal marker, and our data showed that TCD treatment led to a dose- and time-dependent increase in LC3B-II expression (Fig. 5C). In addition, TCD also increased the expression of LC3B-II in MDA-MB-231 cells (Fig. S4). To determine the biological role of autophagy in TCD-induced apoptotic cell death, two autophagy inhibitors, chloroquine (CQ) or bafilomycin A1 (BA) was used in the apoptosis analysis by flow cytometry. As shown in Fig. 5D,E, pre-treatment with CQ in TCD-treated MCF-7 cells increased the proportion of apoptotic cells (annexin V positive) from 14.8% to 27.9% (*P* = 0.000054). Similarly, BA pre-treatment also increased the proportion of apoptotic cells from 14.8% to 46.8% (*P* = 0.0014). Collectively, these data suggested TCD induced protective autophagy but not autophagic cell death in MCF-7 cells.

## Discussion

It has been a long history that people is working on ways to prevent or to fight against cancer by daily foods. Foods with chemopreventive activities include grape, turmeric, apple, and berries^25^. Substantial evidences have demonstrated the wild bitter gourd being not only a functional food, but also with anti-diabetic, anti-inflammatory and anti-tumor activities in various animal studies^12^,^26^,^27^,^28^. It is no wonder that wild bitter gourd is commonly used as a medicine or an adjunct in Asia for a long history^26^. Although reports suggest that triterpenoides, glycoside, saponin, alkaloids, fixed oil, proteins or steroids are probably being responsible for the anti-tumor effect of bitter gourd^6^,^7^,^10^,^29^, the active constituents contributing to the anti-tumor effect of wild bitter gourd have less been characterized clearly. In this study, we demonstrated the antitumor activity of a triterpenoid TCD in breast cancer cells. The identification of a constituent actively against cancer not only supports wild bitter gourd as an anti-tumor food, but also helps future works on producing a new anti-tumor medication, through purification and further modification of the active component.

Our data showed that TCD induced suppression of cell proliferation, caspase-dependent apoptosis, down-regulation of Akt/NF-κB pathway, up-regulation of p38 MAPK and p53 phosphorylation, inhibition of HDACs expression and increased ROS generation in breast cancer cells. The multi-targets action beneficially underlies the anti-tumor effect of TCD in breast cancer. Aberrant signaling networks, including Akt-mTOR, NF-κB, and HDACs, have contributed the acquisition of progression of breast cancer^30^,^31^. Inactivation of p53 also has been implicated in cell invasion of breast cancer^32^. Interestingly, curcumin and sulforaphane, ingredients of tumeric and green tea polyphenols, showed antiproliferative activities in breast cancer through modulation of HDACs^33^,^34^. Additionally, several studies implicated that antioxidants have the potential to intervene the multiple steps involved in breast cancer initiation and progression^35^. Taken together, the pleiotropic mechanism against breast cancer cells highlights the usefulness of wild bitter guard as a practical anti-tumor food.

For cancer therapy, autophagy may accentuate cell death or act as an inhibitor of apoptosis^36^. In our study, we showed that TCD-induced autophagy played a protective role in breast cancer cells under stress. This finding suggests further evaluation of the combination of TCD and an autophagy inhibitor in treating breast cancer.

## Conclusions

The triterpenoid TCD exhibited antiproliferative effect on breast cancer accompanied with induction of apoptosis, down-regulation of Akt-NF-κB signaling, activation of p53 phosphorylation, increased ROS generation and HDAC inhibition. From a clinical perspective, wild bitter gourd extract with enriched TCD quantified by chromatographic fingerprint analysis will provide a better alternative to capsules or tablets for disease management. Further investigations on *in vivo* efficacy of TCD are needed to better understand the role of TCD, alone or in combination with other agent, in breast cancer prevention and treatment.

## Methods

### Plant Materials

TCD was isolated from the whole plant of *M. charantia* L. collected in Pintung County, Taiwan, in October, 2008, and a voucher specimen (2008) has been deposited in the Department of Biological Science and Technology, China Medical University (Taichung, Taiwan). The identity and purity of TCD were verified by proton nuclear magnetic resonance (NMR) spectroscopy, high-resolution mass spectrometry, and 2-D NMR spectrometry using reported spectral data^37^. For *in vitro* experiments, TCD was dissolved in DMSO, and was added to culture medium with a final DMSO concentration of less than 0.1%. Rabbit polyclonal antibodies against various biomarkers were obtained from the following sources: p-^473^Ser-Akt, p-^308^Thr-Akt, PARP, cleavaged caspase-7, caspase-9, LC3B, p-^180^Thr/^182^Tyr-p38 MAPK, p38 MAPK, p-^202^Thr/^204^Tyr-ERK, ERK, p-^15^Ser-p53, p53, HDAC1, HDAC2, HDAC3, HDAC4, acetyl Histone H3, ERα, ERβ, and NF-κB (Cell Signaling Technologies, Beverly, MA); p-^166^Ser-MDM2, MDM2 and Akt (Santa Cruz Biotechnology, Santa Cruz, CA); β-actin (Sigma-Aldrich, St. Louis, MO). The enhanced chemiluminescence (ECL) system for detection of immunoblotted proteins was from GE Healthcare Bioscience (Piscataway, NJ). The GFP-LC3 and peroxisome proliferator-activated receptor response element (PPRE) x3-TK-Luc plasmids were purchased from Addgene (Cambridge, MA). The other chemical and biochemical reagents were obtained from Sigma-Aldrich unless otherwise mentioned.

### Cell Culture

MCF-7 and MDA-MB-231 human breast cancer cells were purchased from the American Type Culture Collection (Manasas, VA). Non-tumorgenic human breast epithelial cell line (H184B5F5/M10) was kindly provided from Dr. Chih-Wen Shu (Kaohsiung Veterans General Hospital). Bone marrow from patients was obtained under a protocol approved by the China Medical University Hospital internal review board. Written informed consent was obtained from all patients in accordance with the Declaration of Helsinki. Normal bone marrow nucleated cells were harvested using Ficoll-PaqueTM PLUS from patients with treatment-naïve non-Hodgkin’s lymphoma for whom bone marrow examination for lymphoma staging was performed but determined to be normal. MCF-7 and MDA-MB-231 human breast cancer cells were maintained in DMEM/F12 contained with 10% fetal bovine serum (FBS) (Gibco, Grand Island, NY), 5 mg/ml of penicillin and 5 mg/ml streptomycin at 37 °C in a humidified incubator containing 5% CO_2_. H184B5F5/M10 cells were maintained in DMEM medium with the same supplements and culture condition.

### Cell Viability Analysis

Effect of TCD on cell viability was assessed using the (MTT) assays^38^ in 6 replicates. Briefly, cells (5 × 10^3^) were seeded and incubated in 96-well, flat-bottomed plates in 10% FBS-supplemented DMEM/F12 for 24 h, and were exposed to TCD at indicated concentrations for different time intervals. The medium was removed, replaced by 200 μL of 0.5 mg/ml MTT in 5% FBS-DMEM/F12, and cells were incubated at 37 °C for 2 h. Medium was removed and the reduced MTT dye was solubilized in 200 μL/well DMSO. Absorbance was determined with a Synergy HT spectrophotometer (Bio-Tek, Winooski, VT, USA) at 570 nm.

### Flow Cytometry

5 × 10^4^ cells were plated and treated with TCD at indicated concentration in 5% FBS-supplemented DMEM/F12 for 72 h. Cells were washed twice in ice-cold phosphate-buffered saline (PBS), and fixed in 70% cold ethanol for 4 h at 4 °C. ROS production was detected using the fluorescence probe 5-(and-6)-carboxy-2′,7′-dichlorodihydrofluoresceindi-acetate (carboxy-DCFDA)^39^. For apoptosis or cell viability evaluation, cells were stained with annexin V and propidium iodide (1 μg/mL) and determined on a BD FACSAria flow cytometer and analyzed by ModFitLT V3.0 software program (Becton Dickinson, Germany).

### Immunoblotting

Drug-treated cells were collected, washed with ice-cold PBS, and resuspended in lysis buffer, consisting of 20 mM Tris-HCl (pH 8), 137 mM NaCl, 1 mM CaCl_2_, 10% glycerol, 1% Nonidet P-40, 0.1% SDS, 100 μM 4-(2-aminoethyl)benzenesulfonyl fluoride, 0.5% deoxycholate, leupeptin at 10 μg/mL, and aprotinin at 10 μg/mL. Soluble cell lysates were collected after centrifugation at 1500*g* for 5 min, and equivalent amounts of protein (60–100 μg) were resolved in 10% SDS-polyacrylamide gels. 15% SDS-polyacrylamide gels were used for the lower molecular weight, LC3B. Bands were transferred to nitrocellulose membranes and blocked with 5% nonfat milk in PBS containing 0.1% Tween 20 (PBST) and incubated overnight with the corresponding primary antibody at 4 °C. After washing with PBST three times, the membrane was incubated at room temperature for 1 h with the secondary antibody with PBST, and visualized by enhanced chemiluminescence.

### HDAC activity assay

Cells (2 × 10^5^/3 mL) were treated with TCD at the indicated concentrations for 24 h. The nuclear isolation kit (Pierce, Rockford, IL) was used according to the manufacturer’s instructions to obtain the nuclear fraction. HDAC activity was determined using a HDAC Fluorometric Activity Assay Kit (Cayman Chemical Company, Ann Arbor, MI, USA) according to the manufacturer’s protocol. Briefly, nuclear extract with or without HDAC inhibitor (1 μM TSA) in duplicate wells, were incubated with an HDAC substrate (200 μM). Deacetylated substrate was measured at 465 nm using a SpectraMax M2 fluorimeter (Molecular Devices, California, USA). Average fluorescence of TSA treated samples was subtracted from the average of untreated corresponding samples. HDAC activity was determined using the deacetylated product concentration obtained using the deacetylated standard curve. HDAC activity is represented as percentage activation of HDAC activity.

### Transient Transfection

Plasmids were transiently transfected into cells by using the Fugene HD reagent (Roche, Mannheim, Germany) according to the manufacture’s protocol. After 24 h, the transfected cells were treated with TCD or DMSO control, and subjected to fluorescent analysis^40^.

### Confocal Imaging

MCF-7 cells expressing GFP-LC3 (2 × 10^5^/3 mL) were seeded in each well of a six-well plate, treated with TCD at the indicated concentration for 30 min. Cells were fixed in 2% paraformaldehyde (Merck) for 30 min at room temperature, and permeabilized with 0.1% Triton X-100 for 20 min. Cells were washed with PBS and then subjected to examination on a Leica TCS SP2 confocal microscope (Leica Biosystems Nussloch GmbH, Heidelberg, Germany) examination.

### Transmission Electron Microscope

Samples were prepared according to an established procedure^41^. Briefly, cells were fixed in a solution containing 2% paraformaldehyde, 2.5% glutaraldehyde, 0.2 M sodium cacodylate for 1 h. Fixed cells were suspended in a buffered solution containing 1% osmic acid for 1 h, followed by dehydration in a graded ethanol series, washing with acetone and embedding into EPON epoxy resin. Ultrathin sections (60–80 nm) were prepared on an ultramicrotome and double-stained with uranyl acetate and lead citrate. All sections were examined and photographed with a Hitachi H-600 transmission electron microscope (Hitachi, Tokyo, Japan).

### Statistical Analysis

All data are presented as means ± S.D. obtained from three independent experiments. Statistical differences were calculated using Student’s *t-test*, with the following symbols of significance level: *P < 0.05, **P < 0.01.

## Additional Information

**How to cite this article**: Bai, L.-Y. *et al*. A triterpenoid from wild bitter gourd inhibits breast cancer cells. *Sci. Rep.* **6**, 22419; doi: 10.1038/srep22419 (2016).

## Supplementary Material

Supplementary Information
