# Supplementary material for: A triterpenoid from wild bitter gourd inhibits breast cancer cells
Source: Sci Rep. 2016 Mar 1;6:22419. doi: 10.1038/srep22419 (PMC4772478; doi:10.1038/srep22419)

SUPPORTING INFORMATION

A triterpenoid from wild bitter gourd inhibits breast cancer cells

Li-Yuan Bai[1,2], Chang-Fang Chiu[2,3], Po-Chen Chu[4,5], Wei-Yu Lin[6], Shih-Jiuan Chiu[7], and Jing-Ru Weng[8,*]

[1]Division of Hematology and Oncology, Department of Internal Medicine; [2]Cancer Center, China Medical University Hospital, Taichung 404, Taiwan, [3]College of Medicine, China Medical University, Taichung 404, Taiwan,[4]Institute of Biological Chemistry, Academia Sinica, Taipei 115, Taiwan, [5]Institute of Basic Medical Sciences, College of Medicine, National Cheng Kung University, Tainan 701, Taiwan, [6]Department of Pharmacy, Kinmen Hospital, Kinmen 891, Taiwan, [7]School of Pharmacy, Taipei Medical University, Taipei 110, Taiwan, [8]Department of Biological Science and Technology, China Medical University, Taichung 404, Taiwan

Figure Legends

Fig. S1. TCD induces apoptosis without increment of PPARγ activity in MDA-MB-231 cells. (A) The dose-dependent effect of TCD on annexin V/PI staining of MDA-MB-231 cells at 72 h. (B) The histogram showing the dose-dependent effect of TCD in MDA-MB-231 cells is representative of three independent experiments. Points, mean; bars, S.D. (n = 3). *$P < 0.05$ compared to the control group. (C) The effect of TCD on PARP cleavage and caspase-9 activation in MDA-MB-231 cells after 72 h exposure. (D) The percentage of apoptotic cells of MDA-MB-231 which was treated with DMSO, or TCD (30 μM) 72 h with or without pre-treatment of 20 μM Z-VAD(OMe)-FMK (Z-VAD) (n = 3). The apoptotic cells were cells with annexin V positive cells in flow cytometric analysis. *$P < 0.05$. (E) Effect of TCD on PPARγ activation in MDA-MB-231 cells. Cells were transfected with PPRE x3-TK-Luc and Renilla plasmids for 24 h before treatment with TCD for 24 h. Data are expressed as the fold change of PPARγ activity in each situation compared to the control. Troglitazone (Trog.) was used as positive control. Values are means ± S.E.M. of three independent experiments. **P*, 0.05 compared to the control group.

Fig. S2. Western blotting analysis of TCD on apoptosis-related biomarkers and HDAC activity in MDA-MB-231 cells. (A) Dose-dependent of TCD on the

phosphorylation and protein expression of Akt, NF-κB, p53, ERα and ERβ. Cells were treated with TCD in 5% FBS-supplemented DMEM/F12 medium for 72h. (B) Effects of TCD on acetyl Histione H3 and HDACs protein expression. (C) TCD inhibited HDAC activity in MDA-MB-231 cells. After treated with DMSO or TCD (25 μM) for 72 h, nuclear extract was used to assess HDAC activity as described in Materials and Methods. (n = 3).

Fig. S3. TCD induced reactive oxygen species (ROS) generation in MDA-MB-231 cells. Left panel, cells were treated with DMSO or 25 μM TCD with or without glutathione (GLU) for 72 h. Right panel, histogram of ROS production of MDA-MB-231 cells treated with DMSO or TCD with or without GLU for 72 h (n = 3). *$P < 0.05$; **$P < 0.01$.

Fig. S4. Dose-dependent effects of TCD on the expression of LC3B-II in MDA-MB-231 cells at 72 h.

Fig. S1

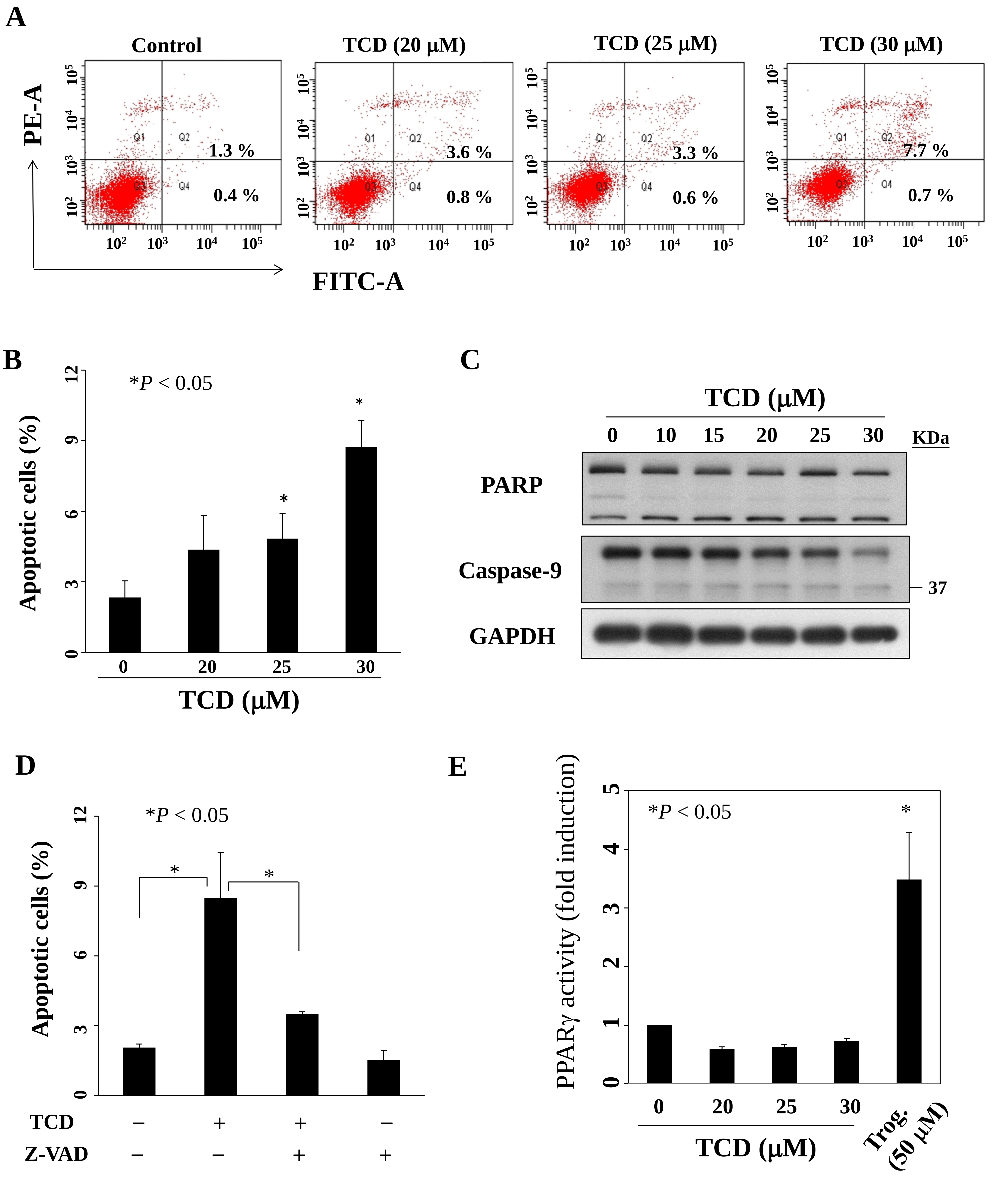
A
Control
TCD (20 μM)
TCD (25 μM)
TCD (30 μM)
PE-A
FITC-A
Q1
Q2
Q3
Q4
1.3 %
0.4 %
3.6 %
0.8 %
3.3 %
0.6 %
7.7 %
0.7 %
B
*P < 0.05
Apoptotic cells (%)
0
20
25
30
TCD (μM)
C
TCD (μM)
0
10
15
20
25
30
KDa
PARP
Caspase-9
37
GAPDH
D
*P < 0.05
Apoptotic cells (%)
TCD
Z-VAD
E
*P < 0.05
PPARγ activity (fold induction)
0
20
25
30
TCD (μM)
Trog. (50 μM)

Fig. S2

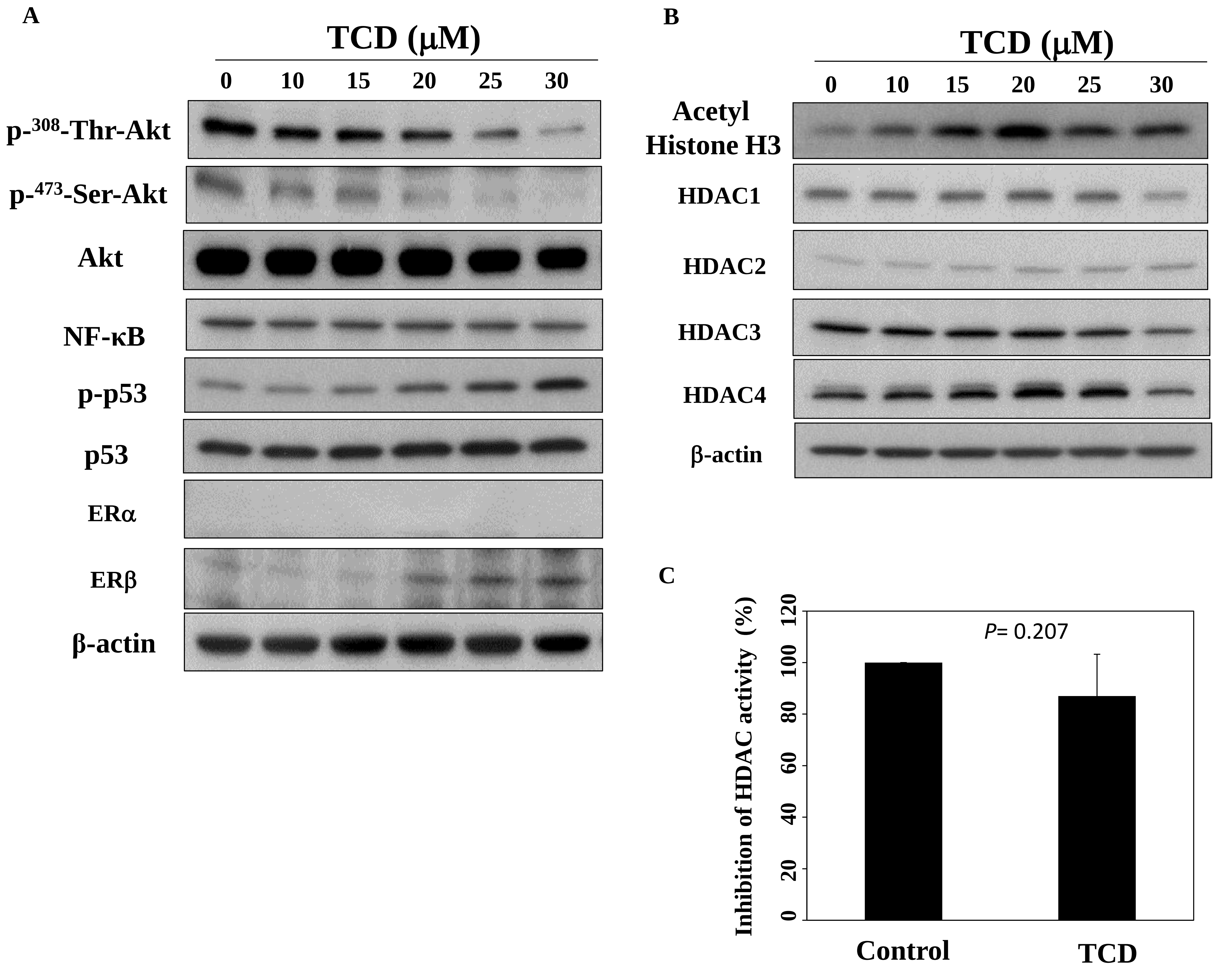
A
TCD (μM)
0 10 15 20 25 30
p-308-Thr-Akt
p-473-Ser-Akt
Akt
NF-κB
p-p53
p53
ERα
ERβ
β-actin
B
TCD (μM)
0 10 15 20 25 30
Acetyl Histone H3
HDAC1
HDAC2
HDAC3
HDAC4
β-actin
C
Inhibition of HDAC activity (%)
0
20
40
60
80
100
120
P= 0.207
Control
TCD

Fig. S3

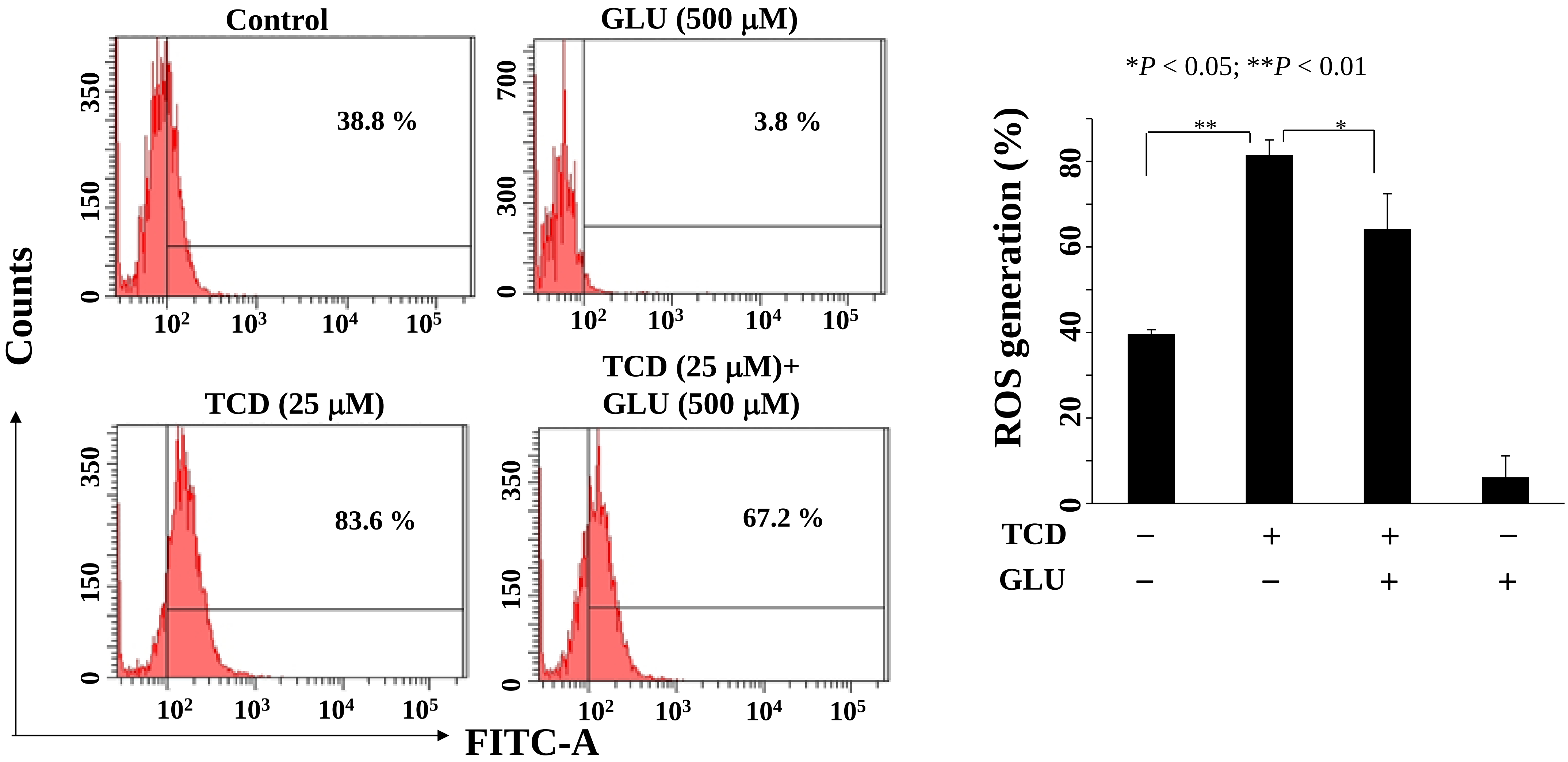
Control
38.8 %
GLU (500 μM)
3.8 %
TCD (25 μM)
83.6 %
TCD (25 μM)+
GLU (500 μM)
67.2 %
Counts
FITC-A
*P < 0.05; **P < 0.01
ROS generation (%)
TCD
GLU

Fig. S4

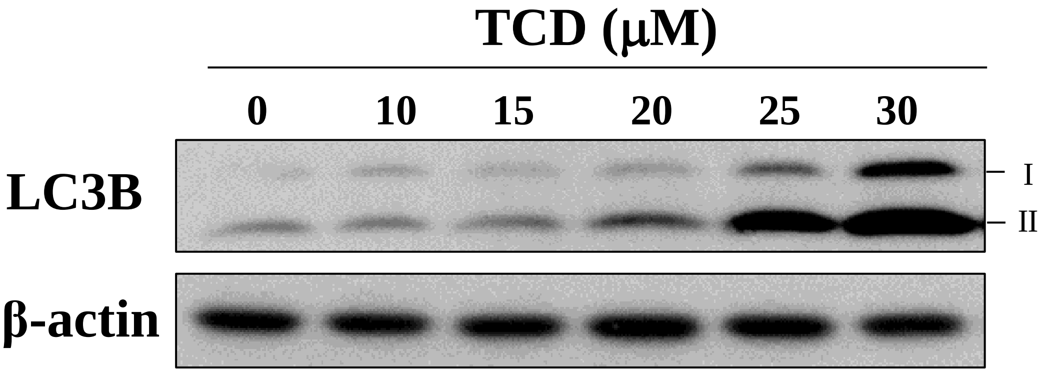

Supplement: Supplementary Information [file srep22419-s1.pdf]
